# Using the Influenza Patient-reported Outcome (FLU-PRO) diary to evaluate symptoms of influenza viral infection in a healthy human challenge model

**DOI:** 10.1186/s12879-018-3220-8

**Published:** 2018-07-28

**Authors:** Alison Han, Jiat-Ling Poon, John H. Powers, Nancy K. Leidy, Ren Yu, Matthew J. Memoli

**Affiliations:** 1 LID Clinical Studies Unit, Viral Pathogenesis and Evolution Section, Laboratory of Infectious Diseases, National Institute of Allergy and Infectious Diseases, National Institutes of Health (NIH), 33 North Drive MSC 3203, Bethesda, MD 20892 USA; 2 Evidera, Bethesda, MD USA; 3 Clinical Research Directorate/Clinical Monitoring Research Program, Leidos Biomedical Research, Inc., NCI Campus at Frederick, Frederick, MD USA

**Keywords:** Influenza, Human challenge study, Symptoms, Virus

## Abstract

**Background:**

In clinical studies involving a healthy volunteer human challenge model, a valid and reliable measure to assess the evolution of patient-reported symptom type and severity following viral exposure is necessary. This study examines the use of the InFLUenza Patient-Reported Outcome (FLU-PRO) diary as a standardized measure of symptom severity in a healthy volunteer human challenge model.

**Methods:**

Healthy adults admitted to the NIH Clinical Center (Day − 1) underwent a 9-day inpatient quarantine after intranasal challenge with a wild-type influenza A/H1N1pdm virus (Day 0). Participants completed the 32-item FLU-PRO diary twice daily for 14 days to assess presence, severity, and duration of symptoms across six body systems. Secondary analyses included descriptive statistics to examine FLU-PRO scores over the course of illness and analysis of variance to compare scores on Day 3 post-challenge by presence of viral shedding, and pre-challenge hemagglutinin and neuraminidase inhibition (HAI and NAI) titers.

**Results:**

All but one subject (99%), who was lost to follow-up, completed twice daily FLU-PRO diaries on all study assessment days. FLU-PRO demonstrated that 61 of 65 subjects reported symptoms (Days: Median 5, Mean 6 ± 7), of whom 37 (61%) had viral shedding. Pre-challenge, 39 (64%) and 10 (16%) subjects had low (< 1:40) HAI and NAI titers, respectively. Nose, throat, body, and gastrointestinal (GI) symptoms reached peak intensity at Day 3, followed by chest/respiratory and eye symptoms at Day 4. Subjects with viral shedding had higher mean FLU-PRO scores compared to those without, except for Eye and GI domains (*p* <0.05). Mean FLU-PRO scores were significantly higher for subjects with low NAI titer (*p* <0.05) across all domains. No significant differences were observed between HAI titer groups. FLU-PRO scores of the low HAI-low NAI group (*n* = 10) were significantly higher (more severe) than the other two groups (*p* < 0.05) (high HAI-high NAI (*n* = 22), low HAI-high NAI (*n* = 29)).

**Conclusions:**

The FLU-PRO had high adherence and low respondent burden. It can be used to track symptom onset, intensity, duration, and recovery from influenza infection in clinical research. In this human challenge study, scores were responsive to change and distinguished known clinical subgroups.

**Trial registration:**

NCT01971255 First Registered October 2, 2013.

**Electronic supplementary material:**

The online version of this article (10.1186/s12879-018-3220-8) contains supplementary material, which is available to authorized users.

## Background

Seasonal influenza is a global public health problem that ranges in severity and can result in hospitalization and death, particularly in high risk groups [1]. Globally, it is estimated that annual seasonal influenza epidemics result in 3 to 5 million cases of severe illness, and 290,000 to 650,000 deaths [1]. This has a significant economic impact, with total economic burden due to annual influenza epidemics estimated at $87.1 billion in the U.S. alone [2].

Healthy volunteer human challenge studies are used to study the pathogenesis of influenza infection, understand the immune response, and gather information on proof-of-concept during the early stages of vaccine or therapeutic development. Although there are no U.S. Food & Drug Administration (FDA) guidelines for defining efficacy endpoints specifically for challenge studies, the FDA guidance for industry “Influenza: Developing drugs for treatment and/or prophylaxis” suggest an efficacy endpoint for influenza drug development trials that would include a combination of measures including patient-reported symptoms [3]. In addition, there are guidance documents to inform the development, selection and use of patient-reported outcome (PRO) measures for use in drug development clinical trials [4] as one way of fostering patient-centered drug development, and properly developed and evaluated PRO instruments provide a unique and important endpoint measure in clinical trials [5]. The InFLUenza Patient-Reported Outcome (FLU-PRO©) measure was developed using methods consistent with these guidelines [6, 7]. The FLU-PRO is a comprehensive, 32-item daily diary that provides a direct measure of the presence and severity of patient-reported influenza symptoms across 6 body systems affected by influenza: Nose, Throat, Eyes, Chest/Respiratory, Gastrointestinal, and Body/Systemic based on evidence from patients regarding the symptoms they find important in influenza. The FLU-PRO total score represents the “severity” of symptoms across these systems as measured by the intensity, frequency, and duration of symptoms.

Qualitative evidence of content validity [6] and data on the reliability, validity, and responsiveness of FLU-PRO scores in hospitalized and non-hospitalized adults with laboratory-confirmed influenza and influenza-like illness have been previously reported, with results suggesting the instrument is a valid measure for evaluating symptoms in these conditions and settings [7, 8].

The FLU-PRO was recently administered in a human influenza challenge study designed to examine the role of hemagglutination inhibition (HAI) and neuraminidase inhibition (NAI) in conferring protection against influenza A virus [9]. In this study, the role of NAI was identified as an important correlate of protection expanding on what has been previously reported [10, 11]. This analysis by Memoli et al. [9] used the same questions as the finalized FLU-PRO instrument, but used an early draft FLU-PRO scoring method since the final FLU-PRO scoring methods and psychometric evaluation were still under development, and FLU-PRO had not previously been used in a healthy volunteer influenza challenge study. Even with the draft scoring method, FLU-PRO still provided valuable data used in the primary analysis. Preliminary secondary analyses were presented at IDWeek 2017 in San Diego, California [12]. This report describes in more detail the analysis of the FLU-PRO data from the same study using the final validated FLU-PRO scoring method in the setting of a human influenza challenge study. It examines the performance of FLU-PRO scores in a healthy volunteer human challenge model, assessing the evolution of symptom frequency, intensity, and duration to define “severity” from exposure to recovery and the relationship between symptom severity and viral shedding, NAI, and HAI titer.

## Methods

### Clinical study

Post-hoc, secondary analyses were performed on data from healthy volunteers enrolled in an influenza challenge study conducted at the National Institutes of Health (NIH) Clinical Center. Study design and primary results were reported previously and adheres to CONSORT guidelines for reporting clinical trials as applicable [9]. Briefly, the study investigated whether participants with a high pre-challenge serum HAI titer were less likely to develop mild to moderate influenza disease after intranasal inoculation with a wild-type influenza A/H1N1pdm virus compared to participants with a low pre-challenge HAI titer.

After signing written informed consent, 65 healthy men and women between 18 to 40 years of age underwent a 9-day inpatient quarantine and intranasal challenge via a nasal atomizer with an infective dose of H1N1pdm and were then monitored in an outpatient clinic periodically over 2 months. Participants were blinded to viral shedding and antibody titer results. Participants received no antiviral medications during the first 7 days post-inoculation and a small number were given other medications to control symptoms if necessary. Participants were tested at baseline to rule out the presence of other viral pathogens. Participants completed the FLU-PRO questionnaire on paper the evening of Day − 1 and morning of Day 0 (pre-inoculation), evening of Day 0 post-inoculation, and twice daily thereafter to Day 14 post-inoculation and then once more on Day 28. The FLU-PRO is a 32-item daily diary assessing influenza signs and symptoms across 6 body systems: Nose (4 items), Throat (3 items), Eyes (3 items), Chest/Respiratory (7 items), Gastrointestinal (4 items), and Body/Systemic (11 items). Respondents are asked to rate each sign or symptom on a 5-point ordinal scale, with higher scores indicating a more frequent sign or symptom. For 27 of the items, the scale is as follows: 0 (“Not at all”), 1 (“A little bit”), 2 (“Somewhat”), 3 (“Quite a bit”), and 4 (“Very much”). For 5 items, severity is assessed in terms of numerical frequency, i.e., vomiting or diarrhea (0 times, 1 time, 2 times, 3 times, or 4 or more times); with frequency of sneezing, coughing, and coughed up mucus or phlegm evaluated on a scale from 0 (“Never”) to 4 (“Always”).

The FLU-PRO total score is computed as a mean score across all 32 items comprising the instrument. Total scores can range from 0 (symptom free) to 4 (very severe symptoms). Six domain scores are also computed, representing symptom severity in each of the assessed body areas. Each domain score is calculated as the mean of all items comprising the domain, with scores ranging from 0 to 4.

### Statistical analyses

The FLU-PRO has been tested as a once-daily measure [7]. In this study, participants completed the diary twice a day. Using paired t-tests, no significant domain or total score differences were observed between the two pre-inoculation administrations (evening Day − 1 and morning Day 0) or the mornings and evenings of Days 1 through 14 post-inoculation (data not shown). Given no added discrimination of a twice daily administration and to be consistent with current use, a daily mean FLU-PRO score was calculated for each participant, taking the average of scores from the two pre-inoculation administrations, and the morning and evening administration of each day post-inoculation. All subsequent analyses used mean daily scores.

Analyses were performed on symptomatic participants only, with asymptomatic participants defined by a FLU-PRO daily total score of zero at every study assessment (Day − 1 to Day 14, and Day 28). Descriptive statistics were used to characterize demographics and clinical characteristics of the study population.

The distributional characteristics of FLU-PRO domain and total scores, including frequency of symptom occurrence within the sample (n, %) and severity of symptoms (score mean (standard deviation, SD), range, floor, ceiling) were examined pre-inoculation, on Day 3 (peak viral shedding and peak number of symptoms), and on Day 10 post-inoculation (by which time most participants were discharged). These days were selected based on viral shedding and number of clinician-assessed symptoms as reported by Memoli et al. [9]. The trajectory of FLU-PRO domain and total scores from Days − 1 to 10 were also plotted and examined.

Analysis of variance (ANOVA) was used to test the ability of FLU-PRO severity scores to differentiate two or more clinical groups. Specifically, domain and total scores on Day 3 were compared by viral shedding status (shedding vs. no shedding), baseline HAI (high vs. low titers), baseline NAI (high vs. low titers), and the combination of baseline HAI-NAI titers (high HAI-high NAI vs. low HAI-high NAI vs. low HAI-low NAI). Day 3 was selected as it was the day of peak viral shedding and peak number of symptoms.

For this set of analyses, assuming the differences between these known-groups would be similar to the difference between influenza positive patients with no/mild symptoms [Mean = 0.98 (SD 0.47)] and moderate symptoms [1.38 (SD 0.57)] [7], the power for detecting significant differences between these known-groups was 0.97.

## Results

### Sample

Sixty-one of the 65 participants (93.8%) were FLU-PRO symptomatic and included in the analyses; 4 subjects were excluded due to FLU-PRO scores of 0 on every entry for the duration of the study. Mean age of these subjects was 28.7 (SD = 6.5), with the majority female (*n* = 33; 54.1%) and white (*n* = 33; 54.1%) (Table 1). At Day 0, 39 participants (63.9%) had low HAI titers, 10 participants (16.4%) had low NAI titers. Thirty-seven participants (60.7%) exhibited viral shedding. Mean days with viral shedding was 2.0 (SD = 2.4; Median = 1; Range: 0–8). No subject received antiviral medications during the acute illness, with only 6 participants receiving oseltamivir after Day 7 post-inoculation. Eight of the 65 participants (12%) received symptomatic treatment with acetaminophen or ibuprofen for fever (*n* = 1), headache (*n* = 5), or menstrual cramps (*n* = 2). Adherence rate for FLU-PRO completion from Days − 1 to 14 was 99%, which was expected to be high as participants were in inpatient quarantine for 9 of the 14 days.

**Table 1:** Sample demographic and clinical characteristics (*N* = 61)

	FLU-PRO Symptomatic (*N* = 61)
Demographic	
Age at inoculation/enrollment, Mean (SD)	28.7 (6.5)
Median (Min-Max)	27.0 (18–47)
Gender, n (%)	
Male	28 (45.9%)
Race, n (%)	
Asian	4 (6.6%)
Black	24 (39.3%)
White	33 (54.1%)
Ethnicity, n (%)	
Hispanic or Latino	7 (11.5%)
Not Hispanic or Latino	54 (88.5%)
Clinical	
HAI Titer, n (%) Low < 1:40	39 (63.9%)
NAI Titer, n (%) Low < 1:40	10 (16.4%)
HAI-NAI Correspondence	
high HAI-high NAI, n (%)	22 (36.1%)
high HAI-low NAI, n (%)	0 (0%)
low HAI-high NAI, n (%)	29 (47.5%)
low HAI-low NAI, n (%)	10 (16.4%)
Viral shedding, n (%) Yes	37 (60.7%)
Number of days with symptoms, Mean (SD)	5.7 (6.8)
Median (Min-Max)	5.0 (0–50)
Number of days with shedding, Mean (SD)	2.0 (2.4)
Median (Min-Max)	1.0 (0–8)

### FLU-PRO symptom prevalence

**Fig. 1:**
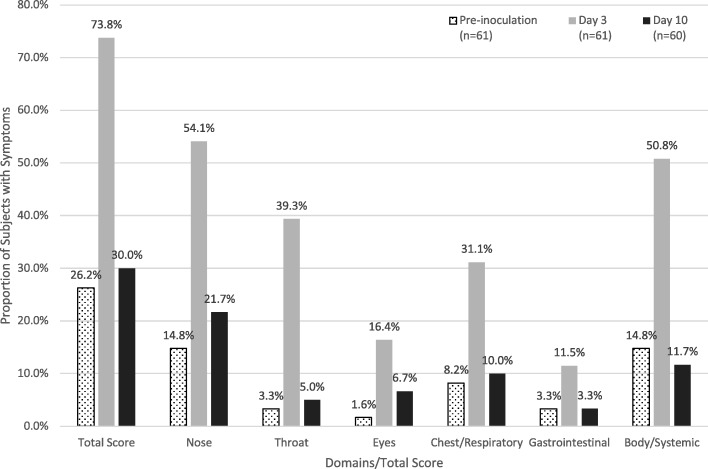
Symptom prevalence by FLU-PRO domains and total scores of symptomatic participants. Proportion of symptomatic participants by FLU-PRO domains and total scores prior to inoculation, Day 3, and Day 10. By Day 3, more participants reported symptoms in all domains and total score compared to baseline with near resolution to baseline prevalence by Day 10

Symptom prevalence by domain pre-inoculation and on Days 3 (peak viral shedding and peak number of symptoms) and 10 is shown in Fig. 1. Pre-inoculation, 8 (13.1%) participants reported symptoms in only one domain and another 8 (13.1%) reported symptoms in two to five domains; no participants reported symptoms in all six domains. On Day 3, 13 (21.3%) participants reported symptoms in only one domain, 30 (49.2%) reported symptoms in two to five domains, and 2 (3.3%) reported symptoms in all 6 domains. By Day 10, ten of sixty (16.7%) participants reported symptoms in one domain, 7 (11.7%) reported symptoms in two to five domains, and no participants reported symptoms in all six domains. Item level descriptive statistics are provided in the online supplement (Additional file 1: Table S1).

### FLU-PRO scores over time

**Fig. 2:**
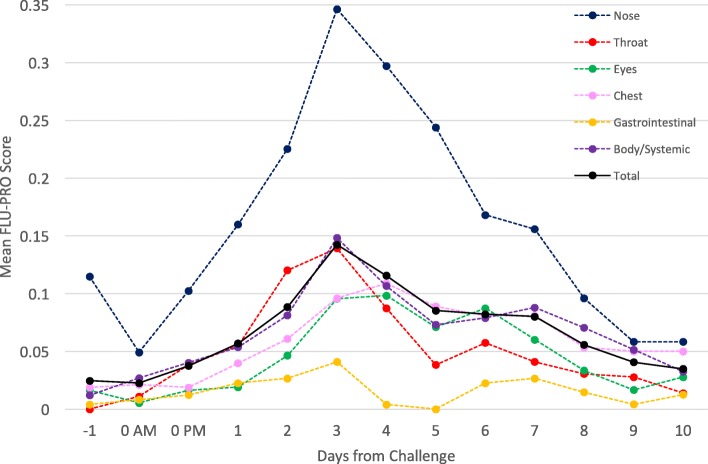
Trajectory of FLU-PRO scores from pre-challenge to Day 10 post-challenge. Mean FLU-PRO scores from Day − 1 (pre-inoculation) through Day 10 by FLU-PRO domains and total scores. Peak FLU-PRO scores occurred on Day 3 for Nose, Body/Systemic, Throat, and Gastrointestinal domains and total score. Peak FLU-PRO scores occurred on Day 4 for Eyes and Chest/Respiratory domains

The trajectory of change in FLU-PRO domain and total scores from Day − 1 to Day 10 are shown in Fig. 2. Pre-inoculation (Day − 1 and Day 0 AM), participants had very low (< 0.05) mean FLU-PRO domains and total score (Fig. 2). Post-inoculation, mean domain and total scores increased, beginning on Day 0 PM and peaking on Day 3 for the Nose, Body/Systemic, Throat, and Gastrointestinal domains and the total score, and on Day 4 for the Eyes and Chest/Respiratory domains before decreasing thereafter. By Day 10, mean scores on all domains and the total score had returned to near pre-inoculation levels (< 0.06).

Throughout the duration of the challenge study, across all 61 subjects, mean domain scores were highest for the Nose domain and lowest for the Gastrointestinal domain. The greatest score fluctuations were observed in the Nose domain with the smallest variation in the Gastrointestinal domain. Symptoms comprising the Nose domain were the most frequently endorsed throughout the study, while Gastrointestinal domain symptoms were the least frequently endorsed.

### FLU-PRO scores by clinical group

**Fig. 3:**
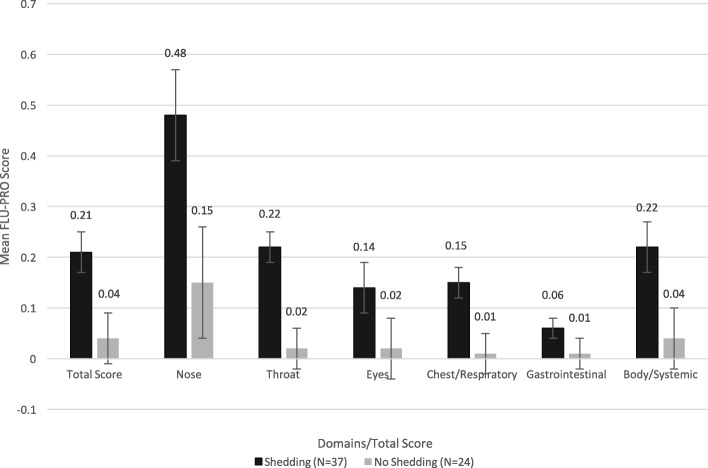
Mean (SE) FLU-PRO domain and total scores on Day 3 by shedding status. Participants with viral shedding had higher scores than those without. The difference was significant for the FLU-PRO total score and the Nose, Throat, Chest/Respiratory, and Body/Systemic domains (*p* < 0.05). Error bars denote standard errors

On Day 3, significant differences in FLU-PRO total score and Nose, Throat, Chest/Respiratory, and Body/Systemic domain scores were found between participants based on viral shedding (all *p* < 0.05; Fig. 3). Participants with viral shedding had significantly higher mean scores than those without on these domains and the total score. For the Eyes and Gastrointestinal domains, participants with shedding also had higher mean scores than those without shedding, although the differences in mean scores were not significant.

**Fig. 4:**
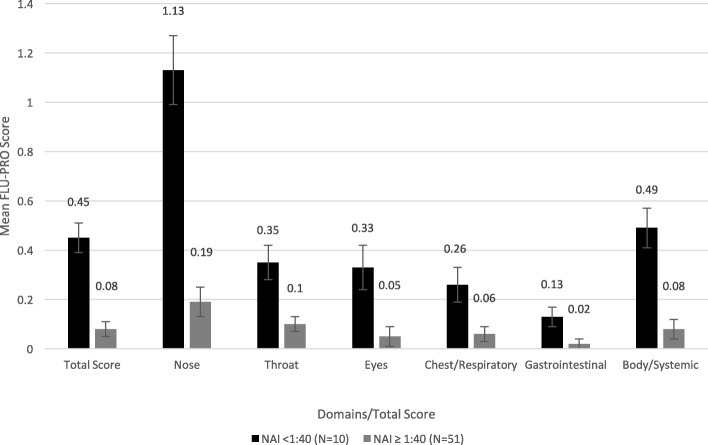
Mean (SE) FLU-PRO domain and total scores on Day 3 by Baseline NAI status. Participants with low NAI titers (< 1:40) had significantly higher mean FLU-PRO domain and total scores compared to those with high NAI titers (all *p* < 0.05). Error bars denote standard errors

**Fig. 5:**
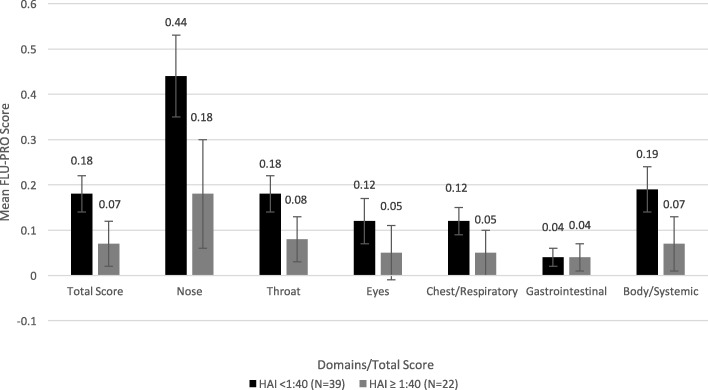
Mean (SE) FLU-PRO domain and total scores on Day 3 by Baseline HAI status. Participants with low HAI titers (< 1:40) had higher mean FLU-PRO domain and total scores than those with high HAI titers, but the differences were not statistically significant. Error bars denote standard errors

Comparing participants by baseline NAI status, on Day 3, participants with low NAI titers (< 1:40) had significantly higher mean FLU-PRO domain and total scores compared to those with high NAI titers (all *p* < 0.05; Fig. 4). However, when comparing participants by baseline HAI status, while those with low HAI titers (< 1:40) had higher mean domain and total scores than those with high HAI titers (Fig. 5), the differences were not statistically significant.

At baseline, 22 participants (36.1%) had high HAI-high NAI, 29 participants (47.5%) had low HAI-high NAI, and 10 participants (16.4%) had low HAI-low NAI (Table 1). No participants had high HAI-low NAI. On Day 3, mean FLU-PRO total and domain scores were highest for the low HAI-low NAI group, followed by the low HAI-high NAI group, with the lowest scores reported by the high HAI-high NAI groups (Table 2). From the ANOVA comparing all three subgroups, there were significant differences in mean scores between subgroups for the FLU-PRO total and all domains scores at *p* < 0.05, except the Gastrointestinal domain (Table 2). Pairwise comparisons using Scheffe’s test identified no significant differences in mean scores between the high HAI-high NAI and the low HAI-high NAI groups. Mean total and domain scores were significantly different at *p* < 0.05 between the high HAI-high NAI and low HAI-low NAI groups, except in the Gastrointestinal domain; scores were also significantly different between the low HAI-high NAI and low HAI-low NAI groups for the total and all domain scores except the Chest/Respiratory and Gastrointestinal domains at *p* < 0.05.

**Table 2:** Mean (SE) FLU-PRO domain and total scores on Day 3 by baseline (Day 0) joint HAI/NAI status

FLU-PRO Total/Domain Score	High HAI – High NAI^a^		Low HAI – High NAI^a^		Low HAI – Low NAI^a^		Overall *F* value (*P* value)^b^	Pairwise Comparison^c^
	N	LS Mean (SE)	N	LS Mean (SE)	N	LS Mean (SE)		
Daily Mean								
Total Score	22	0.07 (0.04)	29	0.09 (0.04)	10	0.45 (0.06)	13.87 (<.0001)	1: 0.9599
								2: < 0.0001
								3: < 0.0001
Nose	22	0.18 (0.10)	29	0.21 (0.08)	10	1.13 (0.14)	17.88 (<.0001)	1: 0.9713
								2: < 0.0001
								3: < 0.0001
Throat	22	0.08 (0.05)	29	0.11 (0.04)	10	0.35 (0.07)	6.07 (0.0040)	1: 0.8095
								2: 0.0055
								3: 0.0148
Eyes	22	0.05 (0.06)	29	0.05 (0.05)	10	0.33 (0.09)	4.14 (0.0209)	1: 0.9962
								2: 0.0437
								3: 0.0292
Chest/Respiratory	22	0.05 (0.04)	29	0.08 (0.04)	10	0.26 (0.07)	3.69 (0.0310)	1: 0.8958
								2: 0.0386
								3: 0.0689
Gastrointestinal	22	0.04 (0.03)	29	0.01 (0.02)	10	0.13 (0.04)	2.65 (0.0792)	1: 0.7754
								2: 0.2510
								3: 0.0794
Body/Systemic	22	0.07 (0.06)	29	0.09 (0.05)	10	0.49 (0.08)	10.49 (0.0001)	1: 0.9666
								2: 0.0004
								3: 0.0004

^a^High HAI - High NAI: HAI > = 1:40 and NAI > = 1:40; Low HAI – High NAI: HAI < 1:40 and NAI > = 1:40; Low HAI – Low NAI: HAI < 1:40 and NAI < 1:40

^b^An analysis of Variance (ANOVA) model

^c^Comparing mean FLU-PRO total/domain scores, 1: group 1 vs. group 2, 2: group 1 vs. group 3, 3: group 2 vs. group 3

## Discussion

This study assessed the ability of the FLU-PRO to quantify the symptoms and severity of influenza in a human challenge study model and to monitor the evolution of symptoms from baseline/pre-challenge through symptom development and resolution. The total score provides an overview of symptom progression and severity overall, while the domain scores show how the severity of symptoms associated with affected body systems vary over time and compare with one another. The symptoms measured by FLU-PRO are due to influenza since other viral pathogens were ruled out at baseline, and participants received minimal symptomatic medications and no antiviral medications during the acute illness.

Mean FLU-PRO scores on peak symptoms day (Day 3) were less severe than values seen in the observational FLU-PRO validation study where naturally-infected subjects were enrolled during a clinic visit, tested for the influenza virus, and recovered at home or in the hospital [7]. The peak mean FLU-PRO total score in naturally-infected patients for the home-recovered group was 1.6 (SD 0.7), with domain scores ranging from 0.6 (SD 0.8) for the GI domain to 1.9 (SD 0.9) for the Body/Systemic domain [7]. Subjects hospitalized with influenza had FLU-PRO total score of 1.4 (SD 0.8), and domain scores ranging from 0.8 (SD 1.0) for the GI domain to 1.9 (SD 0.9) for the Chest/Respiratory domain [7]. The lower scores reported by subjects in the current study may be a function of their youth and health prior to exposure. These subjects had no major comorbidities, while 39% of the natural history study subjects reported one or more comorbidities (Age range: mean age: 40.7 years (SD 16.6), range 18–95 years). Given these challenge participants were younger and healthy, it is likely that their disease represents that of influenza cases that are less likely to be medically attended and therefore not captured in natural history studies. Therefore it is not surprising that the scores were overall lower for these subjects. This provides further support for the sensitivity of the validated FLU-PRO scoring system even among the less symptomatic cases of influenza. In addition, in this human challenge study few concomitant medications were dispensed so resolution of symptoms can be attributed to the natural resolution of influenza infection without the influence of concomitant medications.

Even with the low mean scores/mild symptoms, FLU-PRO total and domain scores were responsive to change from usual (asymptomatic) health to the development of influenza symptoms post-challenge and resolution over time, from Days − 1 to 10. Varied patterns of severity and change were seen across body systems (domains). Applying the FLU-PRO in a challenge setting provides the unique opportunity to better understand symptom onset to resolution since the exact amount and time of exposure are both known. In natural history studies, by the time patients seek care their symptoms may already be quite severe, obviating the ability to capture the early trajectory of their symptoms. In this study of known H1N1pdm exposure, nasal symptoms were more common and more severe, while gastrointestinal symptoms occurred less frequently. Nasal, throat, body/systemic, and gastrointestinal symptoms developed and peaked earlier than chest/respiratory and eye symptoms showing that all symptoms do not occur simultaneously but have an orderly progression over time.

FLU-PRO score differences across viral shedding and both NAI and HAI titer groups offer support for the known-groups validity of FLU-PRO scores [7], provide evidence that the symptoms measured are due to influenza infection and provide evidence for the utility of this tool for use in challenge studies. Participants with viral shedding reported more severe symptoms of influenza than those with no viral shedding. Those with lower NAI titers reported more severe symptoms than those with high NAI titers. In current vaccine development, efficacy has been predominantly evaluated in terms of protection based on HAI titers. The results of more recent research suggest NAI titers have a role in protection that is independent of HAI titers [10, 11]. Symptoms as assessed by the FLU-PRO lasting longer than peak viral shedding also showed the importance of host immune response to symptoms in influenza, and further demonstrated the challenges of using viral shedding as an outcome to define endpoints in influenza studies. Direct measurement of patient symptoms provides the most relevant evidence of patient benefit.

The primary analyses for this challenge study showed that NAI titer was an independent predictor of protection from influenza, and that HAI and NAI titer, jointly considered, could be a better predictor of protection from influenza than either alone [9]. Results of the analyses presented here suggest patient self-report of symptom severity, i.e., the FLU-PRO, can distinguish HAI/NAI titer groups and may be useful as a proxy measure of influenza protection in a clinical or observational study setting. When considered jointly, scores were also able to distinguish between low HAI-low NAI and high HAI-high NAI, and between low HAI-high NAI and low HAI-low NAI groups, again suggesting its applicability for use in a clinical or observational study setting. However, the evidence suggests that at least in the assessment of patient-reported symptoms severity, NAI titer alone would be a better independent predictor of symptoms severity than HAI titer or joint HAI-NAI titers. In addition, symptom measurement adds to the information obtained by titer measurement in evaluating vaccine efficacy, since the evidence shows that in patients who do become infected, their symptoms may be mitigated with an effective vaccine even when disease is not completely prevented.

The FLU-PRO was easy to administer with low respondent burden. Adherence was 99%. Therefore, the FLU-PRO instrument as constructed is suitable for use in clinical trials and observational studies. Once daily administration was sufficient and twice daily administration did not add to the instrument’s discriminative ability.

This was a secondary analysis of existing data, which is a limitation of this report. In addition, the study was not powered for other tests of instrument performance, such as estimates of internal consistency or test-retest reliability. Due to a limited sample size and individual variability, we compared mean scores on pre-inoculation, Day 3, and Day 10. Future challenge studies with a larger sample size would also include longitudinal analysis of individual responses in addition to the group comparisons we have presented. Furthermore, we did not assess patient global impression of return to usual health to define a “responder criteria” for this study, but this is planned for future challenge studies. It is clear that all symptoms do not have to return to scores of zero in order for patients to assess return to their usual state of health [7]. Nevertheless, this is the first time FLU-PRO with a validated scoring system has been used in a human influenza challenge study and it showed responsiveness and known-groups validity in this setting.

## Conclusions

The FLU-PRO is a useful tool with content and construct validity for tracking symptom onset, severity, and recovery from influenza infection in a human challenge study setting. Total and domain scores were responsive to change, with domain scores exhibiting differential patterns of severity and evolution. Differences in symptom severity scores were also observed across known clinical subgroups. Using the FLU-PRO as a standardized measure of influenza symptoms in human challenge studies may facilitate our understanding of the symptomatic evolution of influenza and the effects of new vaccines and drugs to prevent and treat influenza infection.

## Additional file

Additional file 1: **Table S1.** FLU-PRO Item Analysis: Pre-inoculation, Day 3, Day 10. (DOCX 32 kb)
