# Supplementary material for: Using the Influenza Patient-reported Outcome (FLU-PRO) diary to evaluate symptoms of influenza viral infection in a healthy human challenge model
Source: BMC Infect Dis. 2018 Jul 28;18:353. doi: 10.1186/s12879-018-3220-8 (PMC6064178; doi:10.1186/s12879-018-3220-8)
Supplement: Supplementary file 1 — Table S1. FLU-PRO Item Analysis: Pre-inoculation, Day 3, Day 10. (DOCX 32 kb) [file 12879_2018_3220_MOESM1_ESM.docx]

# Additional file

Table S1. FLU-PRO Item Analysis: Pre-inoculation, Day 3, Day 10

|  | Pre-inoculation (N=61) | | | Day 3 (N=61) | | | Day 10 (N=60) | | |
| --- | --- | --- | --- | --- | --- | --- | --- | --- | --- |
|  | Mean ± SD, Median [Range] | Floor N(%) | Ceiling N(%) | Mean ± SD, Median [Range] | Floor N(%) | Ceiling N(%) | Mean ± SD, Median [Range] | Floor N(%) | Ceiling N(%) |
| Runny or dripping nose | 0.0±0.1, 0 [0-1] | 57(93.4%) | 0(0.0%) | 0.3 ± 0.7,  0 [0-3] | 45(73.8%) | 0(0.0%) | 0.0 ± 0.1, 0 [0-1] | 58(96.7%) | 0(0.0%) |
| Congested or stuffy nose | 0.1 ± 0.3,  0 [0-2] | 53(86.9%) | 0(0.0%) | 0.5 ± 0.7,  0 [0-3] | 37(60.7%) | 0(0.0%) | 0.1 ± 0.3, 0 [0-1.5] | 55(91.7%) | 0(0.0%) |
| Scratchy or itchy throat | 0.0 ± 0.1, 0 [0-0.5] | 59(96.7%) | 0(0.0%) | 0.2 ± 0.3,  0 [0-1] | 45(73.8%) | 0(0.0%) | 0.0 ± 0.2, 0 [0-1.5] | 57(95.0%) | 0(0.0%) |
| Sore or painful throat | 0.0 ± 0.0,  0 [0] | 61(100.0%) | 0(0.0%) | 0.2 ± 0.4,  0 [0-2] | 51(83.6%) | 0(0.0%) | 0.0 ± 0.0,  0 [0] | 60(100.0%) | 0(0.0%) |
| Difficulty swallowing | 0.0 ± 0.0,  0.0 [0] | 61(100.0%) | 0(0.0%) | 0.1 ± 0.2,  0 [0-1.5] | 57(93.4%) | 0(0.0%) | 0.0 ± 0.0,  0 [0] | 60(100.0%) | 0(0.0%) |
| Teary or watery eyes | 0.0 ± 0.2,  0 [0-1.0] | 58(95.1%) | 0(0.0%) | 0.1 ± 0.5,  0 [0-3] | 55(90.2%) | 0(0.0%) | 0.0 ± 0.1, 0 [0-1] | 59(98.3%) | 0(0.0%) |
| Sore or painful eyes | 0.0 ± 0.0,  0 [0] | 61(100.0%) | 0(0.0%) | 0.1 ± 0.3,  0 [0-1.5] | 56(91.8%) | 0(0.0%) | 0.1 ± 0.2, 0 [0-1.5] | 57(95.0%) | 0(0.0%) |
| Eyes sensitive to light | 0.0 ± 0.0,  0 [0] | 61(100.0%) | 0(0.0%) | 0.1 ± 0.4,  0 [0-3] | 58(95.1%) | 0(0.0%) | 0.0 ± 0.1, 0 [0-1] | 59(98.3%) | 0(0.0%) |
| Trouble breathing | 0.0 ± 0.0,  0 [0] | 61(100.0%) | 0(0.0%) | 0.1 ± 0.3,  0 [0-2] | 58(95.1%) | 0(0.0%) | 0.0 ± 0.0,  0 [0] | 60(100.0%) | 0(0.0%) |
| Chest congestion | 0.0 ± 0.0,  0 [0] | 61(100.0%) | 0(0.0%) | 0.0 ± 0.1,  0 [0-1] | 60(98.4%) | 0(0.0%) | 0.0 ± 0.1, 0 [0-1] | 59(98.3%) | 0(0.0%) |
| Chest tightness | 0.0 ± 0.0,  0 [0] | 61(100.0%) | 0(0.0%) | 0.0 ± 0.1,  0 [0-1] | 59(96.7%) | 0(0.0%) | 0.0 ± 0.1, 0 [0-1] | 58(96.7%) | 0(0.0%) |
| Dry or hacking cough | 0.0 ± 0.1,  0 [0-1.0] | 60(98.4%) | 0(0.0%) | 0.1 ± 0.4,  0 [0-2] | 54(88.5%) | 0(0.0%) | 0.1 ± 0.4, 0 [0-2] | 58(96.7%) | 0(0.0%) |
| Wet or loose cough | 0.0 ± 0.1,  0 [0-0.5] | 60(98.4%) | 0(0.0%) | 0.0 ± 0.2,  0 [0-1] | 57(93.4%) | 0(0.0%) | 0.1 ± 0.3, 0 [0-2] | 58(96.7%) | 0(0.0%) |
| Headache | 0.0 ± 0.2,  0 [0-1] | 56(91.8%) | 0(0.0%) | 0.4 ± 0.8,  0 [0-2] | 40(65.6%) | 0(0.0%) | 0.1 ± 0.3, 0 [0-2] | 57(95.0%) | 0(0.0%) |
| Head congestion | 0.0 ± 0.1,  0 [0-0.5] | 60(98.4%) | 0(0.0%) | 0.1 ± 0.4 0 [0-2] | 52(85.2%) | 0(0.0%) | 0.0 ± 0.1, 0 [0-1] | 59(98.3%) | 0(0.0%) |
| Sinus pressure | 0.0 ± 0.1,  0 [0-1.0] | 59(96.7%) | 0(0.0%) | 0.2 ± 0.6, 0 [0-3.5] | 51(83.6%) | 0(0.0%) | 0.0 ± 0.2, 0 [0-1] | 57(95.0%) | 0(0.0%) |
| Felt dizzy | 0.0 ± 0.0,  0 [0] | 61(100.0%) | 0(0.0%) | 0.0 ± 0.2 0 [0-1.5] | 58(95.1%) | 0(0.0%) | 0.0 ± 0.0,  0 [0] | 60(100.0%) | 0(0.0%) |
| Lack of appetite | 0.0 ± 0.1,  0 [0-0.5] | 60(98.4%) | 0(0.0%) | 0.1 ± 0.3, 0 [0-1.5] | 53(86.9%) | 0(0.0%) | 0.0 ± 0.1, 0 [0-1] | 58(96.7%) | 0(0.0%) |
| Felt nauseous | 0.0 ± 0.0,  0 [0] | 61(100.0%) | 0(0.0%) | 0.1 ± 0.3,  0 [0-1.5] | 58(95.1%) | 0(0.0%) | 0.0 ± 0.0,  0 [0] | 60(100.0%) | 0(0.0%) |
| Stomach ache | 0.0 ± 0.1,  0 [0-0.5] | 60(98.4%) | 0(0.0%) | 0.1 ± 0.3,  0 [0-1.5] | 56(91.8%) | 0(0.0%) | 0.0 ± 0.3, 0 [0-2.5] | 59(98.3%) | 0(0.0%) |
| Sleeping more than usual | 0.0 ± 0.1,  0 [0-0.5] | 59(96.7%) | 0(0.0%) | 0.1 ± 0.3,  0 [0-1.5] | 51(83.6%) | 0(0.0%) | 0.1 ± 0.4, 0 [0-2.5] | 55(91.7%) | 0(0.0%) |
| Body aches or pains | 0.0 ± 0.3,  0 [0-2] | 59(96.7%) | 0(0.0%) | 0.3 ± 0.6, 0 [0-2] | 48(78.7%) | 0(0.0%) | 0.1 ± 0.5, 0 [0-3.5] | 57(95.0%) | 0(0.0%) |
| Weak or tired | 0.0 ± 0.1,  0 [0-0.5] | 60(98.4%) | 0(0.0%) | 0.1 ± 0.3, 0 [0-1.5] | 52(85.2%) | 0(0.0%) | 0.1 ± 0.3, 0 [0-1.5] | 58(96.7%) | 0(0.0%) |
| Chills or shivering | 0.0 ± 0.3,  0 [0-2] | 60(98.4%) | 0(0.0%) | 0.1 ± 0.2, 0 [0-1] | 55(90.2%) | 0(0.0%) | 0.0 ± 0.0,  0 [0] | 60(100.0%) | 0(0.0%) |
| Felt cold | 0.0 ± 0.2,  0 [0-1] | 58(95.1%) | 0(0.0%) | 0.2 ± 0.4, 0 [0-2] | 49(80.3%) | 0(0.0%) | 0.0 ± 0.1, 0 [0-0.5] | 58(96.7%) | 0(0.0%) |
| Felt hot | 0.0 ± 0.1,  0 [0-0.5] | 59(96.7%) | 0(0.0%) | 0.1 ± 0.3, 0 [0-1.5] | 55(90.2%) | 0(0.0%) | 0.0 ± 0.1, 0 [0-0.5] | 58(96.7%) | 0(0.0%) |
| Sweating | 0.0 ± 0.0,  0 [0] | 61(100.0%) | 0(0.0%) | 0.1 ± 0.3, 0 [0-1.5] | 58(95.1%) | 0(0.0%) | 0.0 ± 0.0,  0 [0] | 60(100.0%) | 0(0.0%) |
| How many times did you vomit | 0.0 ± 0.1,  0 [0-0.5] | 60(98.4%) | 0(0.0%) | 0.0 ± 0.0, 0 [0] | 61(100.0%) | 0(0.0%) | 0.0 ± 0.0,  0 [0] | 60(100.0%) | 0(0.0%) |
| How many times did you have diarrhea? | 0.0 ± 0.1,  0 [0-0.5] | 60(98.4%) | 0(0.0%) | 0.0 ± 0.2, 0 [0-1] | 59(96.7%) | 0(0.0%) | 0.0 ± 0.1, 0 [0-0.5] | 59(98.3%) | 0(0.0%) |
| Sneezing | 0.2 ± 0.3,  0 [0-1.5] | 47(77.0%) | 0(0.0%) | 0.4 ± 0.7, 0 [0-3] | 39(63.9%) | 0(0.0%) | 0.1 ± 0.3, 0 [0-1.5] | 51(85.0%) | 0(0.0%) |
| Coughing | 0.1 ± 0.2,  0 [0-1] | 52(85.2%) | 0(0.0%) | 0.2 ± 0.6, 0 [0-3] | 46(75.4%) | 0(0.0%) | 0.1 ± 0.4, 0 [0-2] | 55(91.7%) | 0(0.0%) |
| Coughed up mucus or phlegm | 0.0 ± 0.2,  0 [0-1.0] | 59(96.7%) | 0(0.0%) | 0.2 ± 0.5,  0 [0-2.5] | 53(86.9%) | 0(0.0%) | 0.1 ± 0.3, 0 [0-2] | 55(91.7%) | 0(0.0%) |
